# Immune checkpoint inhibitor therapy associated with IgA nephropathy: a case report and literature review

**DOI:** 10.3389/fimmu.2024.1393901

**Published:** 2024-05-14

**Authors:** Melchior Chabannes, Ziriab Lisri, Stéphane Lang, Jean Seibel, Guillaume Eberst, Didier Ducloux, Céline Pursun, Marie Agnes Dragon Durey, Marie-Alexandra Alyanakia, Sophie Felix, Thomas Crepin

**Affiliations:** ^1^ University Hospital, Besançon, Department of Nephrology, Dialysis and Renal Transplantation, Besancon, France; ^2^ Université de Franche-Comté, CHU Besançon, EFS, INSERM, UMR RIGHT, Besançon, France; ^3^ University Hospital, Besançon, Department of Pneumology, Besancon, France; ^4^ Department of Biological Immunology, Hôpital Européen Georges Pompidou, Assistance Publique-Hôpitaux de Paris, Paris, France; ^5^ INSERM UMRS 1138, Cordelier Research Center, Paris, France; ^6^ Université de Paris Cité, Paris, France; ^7^ Service d’Immunologie Biologique, Hôpital Necker-Enfants Malades, Assistance Publique- Hôpitaux de Paris (AP-HP), Université de Paris, Paris, France; ^8^ University Hospital, Besançon, Department of Pathology, Besancon, France

**Keywords:** IgA nephropathy, IgAN, immune check inhibitor (ICI), pembrolizumab, glomerulonephritis, immune-related adverse events (IRAE), AKI (acute kidney injury)

## Abstract

Immune checkpoint inhibitors (ICIs) dramatically improve the prognosis of many malignancies but at the cost of numerous side effects, which may limit their benefits. Acute kidney injury associated with immune checkpoint inhibitors most frequently are acute tubulointerstitial nephritis (ATIN), but various cases of glomerulonephritis have also been reported. Herein, we report a case of severe IgA nephropathy (IgAN) associated with ICIs and carry out a literature review. IgAN was diagnosed in a median time of 5 months (range 1–12 months) after the initiation of ICIs, with heterogeneous severity, and usually treated by corticosteroid and discontinuation of ICIs. In contrast to our case, renal outcomes in literature were often favorable, with recovery of renal function and a reduction in proteinuria on treatment. Although IgAN related to ICIs is a much rarer complication than ATIN, it may still be underdiagnosed. Careful questioning and screening for asymptomatic hematuria should be performed before using ICIs.

## Introduction

Onco-nephrology is an emerging discipline for multiple reasons. Chronic kidney disease leads to a greater risk of cancer. Conversely, several oncological treatments or paraneoplastic syndromes may result in renal injury (1). More recently, the discovery and understanding of the role of immune checkpoint in T-cell activation and function, and of the way cancer cells hijack this system, have been a major progress in the field of oncology. Immune checkpoint inhibitors (ICIs) such as PD1 (*e.g.*, nivolumab or pembrolizumab), programmed death-protein-ligand PD-L1 (*e.g.*, durvalumab), or cytotoxic T lymphocyte-associated protein 4 CTLA-4 (*e.g.*, ipilimumab) inhibitors have dramatically improved prognosis of cancers and are widely used. Despite their efficiency, ICIs are associated with a wide spectrum of autoimmune-related toxicities called immune-related adverse events (irAEs). The most affected organs are the skin, the gastrointestinal tract, and the liver ranging from 54% to 76% of cases (2). Acute kidney injury related to ICIs (ICI-AKI) is less common, ranging from 2% to 4.9% (2). Renal side effects of ICIs are mostly represented by acute tubulointerstitial nephritis (ATIN). Gupta et al. (3) have described an international retrospective cohort of 429 patients with ICI-AKI. Among them, 151 underwent kidney biopsy and 125 (82.7%) showed ATIN lesions. However, glomerulonephritis (GN) is also reported (2), and numerous glomerular lesions are described as well. In a retrospective study from Mamlouk et al. (4), 16 cases of ICI-AKI were reported among 6,412 patients. Of these, 14 had ATIN, which could be isolated in only five patients; other lesions were found such as pauci-immune GN, IgA nephropathy (IgAN) (two cases), membranous nephropathy, C3 GN, focal segmental glomerulosclerosis, and AA amyloidosis. In a recent meta-analysis by Kitchlu et al. (5), of 45 kidney biopsies demonstrating ICI-related glomerular disease, the most common GN was proliferative GN (pauci-immune and anti-GBM GN n = 15, C3 GN n = 5), immune-complex GN n = 2, and lupus-like nephritis n = 1, which accounted for half of the cases. The second most common (35%) was nephrotic syndromes such as podocytopathies (n = 11), AA amyloidosis (n = 4), and membranous nephropathy (n = 1). Only four cases (8.9%) were IgAN (5).

The following case report presents the case of a patient with severe IgA nephropathy, to highlight the difficulties of managing these patients and the need for close collaboration between oncology and nephrology teams.

## Case report

**Figure 1:**
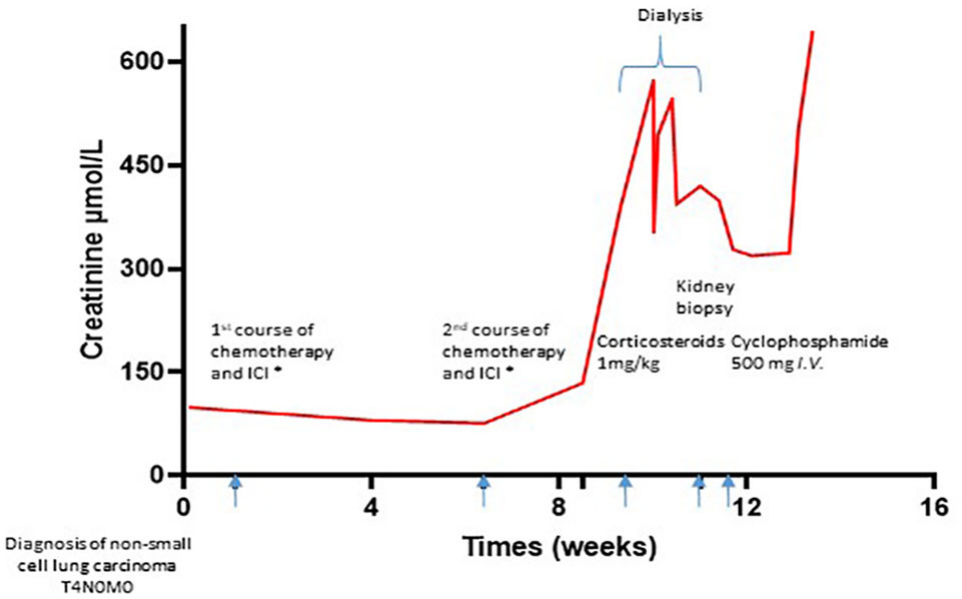
Timeline. Chemotherapy and ICI consisting of carboplatin AUC 6, paclitaxel 200 mg/m², and pembrolizumab 200 mg IV every 3 weeks. ICI, immune checkpoint inhibitor.

A 65-year-old man was diagnosed with non-small cell lung carcinoma (NSCLC) T4N0M0 and treated by chemotherapy and ICI infusion including carboplatin AUC 6, paclitaxel 200 mg/m², and pembrolizumab (a humanized monoclonal antibody against programmed death protein {PD-1}) 200 mg IV every 3 weeks. Three weeks after the second course of his treatment, he developed a severe acute kidney injury with an increase in creatinine from 108 µmol/L to 570 µmol/L in 1 week, associated with oliguria at 300 mL per day. The CT scan showed kidneys without morphological abnormalities or obstructive uropathy. AKI required two sequential dialysis sessions. He reported having microscopic hematuria a long time ago without any investigation. He had no history of chronic kidney disease, high blood pressure, urological disease, and gross hematuria (**Figure 1**).

**Figure 2:**
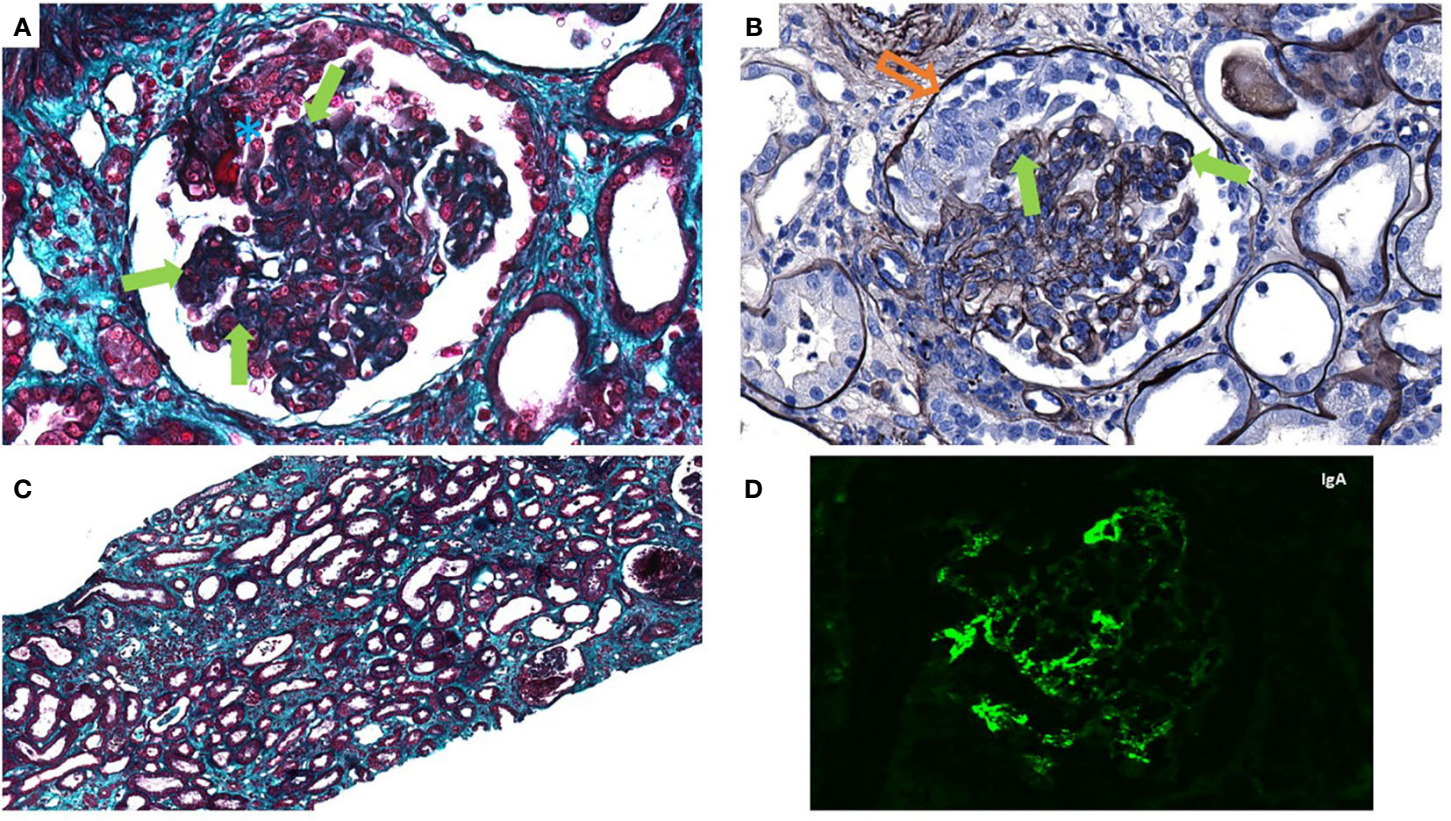
The patient’s kidney biopsy shown by light microscopy: Glomeruli showing endocapillary hypercellularity (solid arrow, green), cellular crescent (hollow arrow, orange), and fibrinoid necrosis (asterisk, blue), ×40 magnification, Masson’s trichrome stain **(A)** and Jones’ silver stain **(B)**. Interstitium showing mild to moderate inflammation, edema, and diffuse acute tubular injury **(C)**, ×10 magnification, Masson’s trichrome stain. **(D)** Immunofluorescence showed IgA mesangial staining without other significant deposit.

The patient presented an impure nephrotic syndrome with lower-limb edema, a urinary protein/creatinine ratio of 3.4 g/g, and serum albumin at 18 g/L, associated with microscopic hematuria (1129/mm^3^). There were no extra-renal manifestations. Immune testing revealed normal complement and negative anti-neutrophil cytoplasmic (ANCA) and anti-glomerular basement membrane antibodies (anti-GBM) of the IgG isotype. Serum protein electrophoresis was consistent with an inflammatory profile. Treatment with oral corticosteroids (1 mg/kg) was introduced in the context of rapidly progressing glomerulonephritis and the use of ICIs. The kidney biopsy, performed 7 days after the admission and the beginning of oral corticosteroid, contained 16 glomeruli (**Figure 2**). By light microscopy, we observed intense proliferative lesions consisting of mesangial hypercellularity, endocapillary proliferation in 80% of glomeruli, and five active glomerular crescents. Glomerular lesions were associated with interstitial inflammation with inflammatory elements but not enough to assess acute interstitial nephritis. By immunofluorescence, we observed intense mesangial IgA deposit with co-deposition of C3 but no fibrinogen deposit. A diagnosis of severe IgAN was made with MEST-C [i.e., a prognostic histological score (6)] scoring M1E1S0T1-C2. Therefore, chemotherapy and ICI therapy were discontinued, and immunosuppressive therapy was intensified with a pulse of cyclophosphamide (500 mg *i.v.*) in addition to the corticosteroid. Cyclophosphamide was stopped due to gross hematuria and radiotherapy initiation. Unfortunately, despite an improvement in renal function with cessation of dialysis 7 days after the start of treatment, the patient died 4 weeks later from a severe lung infection and multiple-organ failure due to the progression of the lung carcinoma.

## Discussion

Although IgAN is the most common primary GN in the world (7), only a few cases secondary to ICI treatment have been described. In the literature, we found 11 case reports (nine IgAN and two IgA vasculitis), which are briefly described in **Table 1** [no clinical data are available in the four cases described by Kitchlu et al. (5)]. We excluded two cases, one due to the absence of histologic evidence (17) and the second because the final diagnostic was postinfectious GN (8). Nine were case reports and two cases were described by Mamlouk et al. (4) among 16 patients with ICI-induced irAEs. Patients were mostly male with a median age of 70 (range 50–78) years old, with no previous history of kidney disease. ICI-AKI occurred in a median time of 5 months (1 to 12 months) after the beginning of ICIs. Renal manifestations consisted of glomerular syndrome with proteinuria and microscopic hematuria in almost every case, associated with different severity levels of AKI. Histopathologic kidney biopsy analysis showed typical IgAN features in nine cases (9–14, 16) and IgA vasculitis in two cases (15, 18) associated with mild ATIN in seven cases. In our case, patient characteristics were similar to that of the literature. Still, AKI appeared earlier after ICI initiation, and histopathologic features were much more severe with intense endocapillary proliferation and active crescentic GN. He also had nephrotic syndrome, which was not present in any of the other cases. Nephrotic syndromes are not a typical feature of IgAN and are often associated with segmental sclerosis. Cases of minimal change disease (MCD) associated with IgAN have also been described (19). In our case, there was no segmental sclerosis and no amyloidosis, and we did not perform electromicroscopy for confirmation of MCD. Other factors may have amplified the hypoalbuminemia, such as a chronic inflammatory syndrome associated with cancer and a recent lung infection.

**Table 1:** Clinical presentation of different IgA nephropathy related to immune checkpoint inhibitor.

	Our case	Mamlouk et al., 2016 (2 cases) (4)	Kishi et al., 2018 (8)	Wang et al., 2020 (9)	Oki et al., 2020 (10)	Mitarai et al., 2022 (11)	Tanabe et al., 2020 (12)	Dougherty et al., 2021 (13)	Belkaid et al., 2020 (14)	Casafont-Solé et al., 2020 (15)	Palamaris et al., 2022 (16)
**Age/gender**	65/M	1) 69/M 2) 50/W	72/M	72/M	75/W	70/M	78/M	70/W	70/M	64/M	73/M
**Basal kidney function serum creatinine (mg/dL)/eGFR (mL/min/1.73 m²)**	0.9/89	1) 1.4/51 2) 0.8/86	NA/69	1.15/47	0.65/87	NA/70	0.64/93	0.8/64	NA No renal history	NA No renal history	NA
**Others irAEs**	–	– -	–	Adrenal insufficiency		–	–	Malar-like rash	Arthralgia, purpura, and digestive disorder	Inflammatory rheumatism,	–
**Cancer type**	Lung NSCLC	1) Uveal melanoma 2) Melanoma	Lung squamous cell carcinoma T3N2M0.	Lung mesothelioma T4N2M0	Lung NSCLC	Lung squamous cell carcinoma	Gastric cancer T3N3M1	Lung NSCLC stage IV	metastatic melanoma	Lung Squamous cell carcinoma pT1aN1M0	Bladder Cancer
**Drug/lines**	Pembrolizumab/I line	1) Nivolumab + ipilimumab 2) Pembrolizumab	Nivolumab/I line	Pembrolizumab/II line	Pembrolizumab/II line	Pembrolizumab/II line	Nivolumab/III Line	Ipilimumab/II line Prior use of pembrolizumab in I line	Nivolumab + ipilimumab/II line	Durvalumab/I line	Atezolizumab/NA
**Delay before ICI-AKI since initiation of ICIs (delay before ICI-AKI since the last course of ICIs)**	8 weeks (3 weeks)	1) 6 weeks (NA) 2) 12 weeks (NA)	24 weeks (NA)	20 weeks (NA)	36 weeks (NA)	8 weeks (NA)	32 weeks (12 weeks)	4 weeks (4 weeks)	15 weeks (3 weeks)	20 weeks (NA)	48
**Renal presentation**	Nephrotic syndrome and AKI Stage 3^§^	1)AKI stage 2 2) AKI stage 3	AKI	AKI stage 1	Glomerular syndrome ^§§^/AKI grade 2 (CTCAE)	Glomerular syndrome/AKI	Glomerular syndrome and AKI Stage 3	Glomerular syndrome/AKI stage 3	Glomerular syndrome	Glomerular syndrome, no AKI	AKI
**Creatinine level (mg/dL)**	4.77	1) 2.40 2) 3.08	1.35	1.57	0.79	NA^§§§^	1.45	2.5	0.94	NA	2
Proteinuria (g/g)/Hematuria^*^	3.4/+	1) 7.7/+ 2) −/−	1.69/+	−/−	1.7/+	3.0/+	3.59/+	3+ (dipstick)/+	1/+	0.539/+	NA/+
**Oxford MEST-C score**	M1E1S0T1-C2	1) M?E1S1T0-C0 (according to biopsy description) 2)NA Focal segmentally sclerotic glomeruli. No active lesion	NA Mesangial proliferation WO endocapillary nor crescent described (M1E0C0)?	M1E0S0T0C0	NA Mesangial and endocapillary proliferation. No crescent described (M1E1C0 )?	M0S0E1T2C1 Fibrotic crescent	M1S0E0T1C1	NA Mesangial and endocapillary hypercellularity, 5 cellular crescents	NA Mesangial and endocapillary proliferation	NA	M1E0S0T1-C0
**Associated ATIN**	+/−	1) +mild 2) + mild	–	–	+	+	+ focal	–	–	NA	Mild
Treatment ^**^	ICI discontinuation + CTS I.V. bolus then 1 mg/kg rapid tapering + CYP	1) ICI discontinuation + oral CTS at.0.5 mg/kg 2) ICI discontinued + oral CTS 2 mg/kg + MMF 1 g + IBD infliximab (1 dose)	ICI discontinuation	ICI 2months discontinuation	ICI discontinuation	CTS I.V. bolus then 1 mg/kg rapid tapering	ICI discontinuation + oral CTS 0.6 mg/kg rapid tapering	ICI discontinuation + CTS I.V 1 mg/kg rapid tapering	ICI discontinuation + CTS I.V. bolus then 1mg/kg rapid tapering	ICI discontinuation + CTS 0,5mg/Kg	NA
**Outcomes** **(details if available)**	Death	1) Renal recovery followed by relapsed 2) Renal recovery	Renal recovery at 4 months	No renal recovery (persistent CKD)	Renal recovery (proteinuria decreased after 6 months)	Renal recovery (decreased in proteinuria and creatinine)	No renal recovery (increased creatinine level at 1.82 mg/dL)	No renal recovery	Renal recovery (NA)	Renal recovery (NA)	NA

*Always microscopic hematuria.

**Treatment always included symptomatic treatment.

^§^Glomerular syndrome: high blood pressure in association with proteinuria and/or hematuria.

^§§^Staging based on Kidney Disease: Improving Global Outcomes definition.

^§§§^Decrease of the eGFR to 55 mL/min/1.73 m².

Conversion factors for units: serum creatinine in mg/dL to µmol/L, ×88.4.

irAEs, immune-related adverse events; CKD, chronic kidney disease, CTCAE, common terminology criteria for adverse events; AKI, acute kidney injury; CYP, cyclophosphamide; IBD, inflammatory bowel disease; MMF, mycophenolate mofetil; NA, no available; IV, intravenous; CTS, corticosteroid; ESKD, end-stage kidney disease; Vs, versus.

- mean negative or absent; + mean positive or present.

Mechanisms of irAEs are not completely understood, including for ICI-AKI mechanisms. Common hypotheses are 1) aberrant self-reactive T-cell activation and 2) loss of peripheral tolerance toward an intrinsic kidney antigen (2). Most patients with ICI-AKI had concomitant medication, and the use of proton pump inhibitors was proven to be a major risk factor for developing ICI-ATIN (2, 3). These results supported the hypothesis of a loss of peripheral tolerance. The patient was taking PPIs and NSAIDs and had received antibiotics in the previous weeks. Although these drugs have a strong association with the risk of ATIN (3), their role in the development of glomerulonephritis has not yet been established. IgAN is a multiple-hit disease (6) in which aberrantly galactosylated IgA1 acts as autoantigens that induce the production of IgG antibodies forming immune complexes that are deposited in the mesangium of the kidney and triggering an inflammatory reaction within the kidney. Recently, Nihei et al. (20) demonstrated the presence of IgA autoantibodies directed against a protein expressed on the membrane surface of mesangial cells in 30% of patients with IgAN. This mechanism, which remains to be confirmed, may be part of the breakdown in peripheral tolerance induced by ICIs. As reported, ICI-induced IgAN is very rare; however, its incidence may be underestimated because IgAN can be paucisymptomatic, with only microscopic hematuria and a low proteinuria level. The role of renal biopsy in diagnosing ICPI-AKI is controversial and currently debated (21). In our opinion, these data highlight the need for renal monitoring before, during, and after ICI therapy and the importance of rapidly performing a kidney biopsy in AKI situations. In our case, the patient probably had a history of renal disease, but this appeared only after careful questioning in our department. He reported positive dipstick to microscopic hematuria, screened at occupational medicine that had never been explored. We hypothesize that the patient had preexisting subclinical IgAN and became symptomatic after exposure to ICIs due to a rupture of peripheral tolerance. Alternatively, the severity of the lesions, particularly glomerular crescents, led us to hypothesize another autoimmune mechanism. ANCA of IgA class (ANCA-IgA) has previously been described in crescentic GN with IgA deposition (22). Therefore, we performed an ANCA-IgA ELISA before and after the use of pembrolizumab. Both tests were negative in specific IgA anti-MPO and -PR3.

There is no strong recommendation for ICI-AKI management. ICI discontinuation and rapid introduction of oral corticosteroids are recommended in the case of ICI-AKI for grade 2 or more ATIN (2, 23). In the case of glomerular disease, immunosuppressive therapy could be considered. In our case, crescentic GN and the severity of AKI justified the initiation of immunosuppressive treatment with a high dose of corticosteroids and one pulse of cyclophosphamide. Cyclophosphamide was discontinued during treatment due to the progression of lung cancer requiring radiotherapy and hemorrhagic cystitis. Given the severity of renal damage, we did not attempt a rechallenge with ICIs. In the reviewed literature, 8/11 patients received corticosteroids without a consistent regimen; one patient also received mycophenolate mofetil and anti-TNF-a therapy in an inflammatory bowel disease context. Treatments for IgAN are currently undergoing a revolution, with many molecules showing significant results in both symptomatic (e.g., sparsentan or dapagliflozin) and specific treatment (e.g., Nefecon, a targeted-release formulation of budesonide) (24, 25). The use of these new treatments in the very specific context of immunotherapy remains to be explored.

In conclusion, we report a severe case of crescentic IgAN related to the use of pembrolizumab. IgAN related to ICIs is a much rarer complication than ATIN but could be underdiagnosed. All these cases call for a close collaboration between oncologists and nephrologists.

## Data availability statement

The original contributions presented in the study are included in the article/supplementary material. Further inquiries can be directed to the corresponding author.

## Ethics statement

Written informed consent was obtained from the individual(s) for the publication of any potentially identifiable images or data included in this article.

## Author contributions

MC: Conceptualization, Methodology, Writing – original draft, Writing – review & editing. ZL: Writing – original draft, Writing – review & editing. SL: Supervision, Writing – review & editing. JS: Writing – review & editing, Writing – original draft. GE: Supervision, Validation, Writing – review & editing, Writing – original draft. DD: Supervision, Validation, Writing – review & editing, Writing – original draft. CP: Writing – review & editing, Writing – original draft. MD: Investigation, Supervision, Writing – review & editing, Writing – original draft. MA: Supervision, Writing – review & editing, Writing – original draft. SF: Supervision, Writing – review & editing, Writing – original draft. TC: Methodology, Supervision, Validation, Writing – original draft, Writing – review & editing.
